# EHMTI-0362. Non-invasive vagus nerve stimulation with gammacore® for prevention and acute treatment of chronic cluster headache: report from the extension phase of the preva study

**DOI:** 10.1186/1129-2377-15-S1-I8

**Published:** 2014-09-18

**Authors:** C Gaul, H Diener, K Solbach, N Silver, A Straube, D Magis, U Reuter, A Andersson, EJ Liebler

**Affiliations:** 1 Migraine and Headache Clinic, University of Duisburg-Essen, Königstein, Germany; 2 Department of Neurology, University Hospital Essen, Essen, Germany; 3 Department of Neurology, Walton Centre for Neurology and Neurosurgery, Liverpool, UK; 4 Department of Neurology, Ludwig Maximilian University of Munich, Munich, Germany; 5 Department of Neurology, Liège University, Liège, Belgium; 6 Berlin NeuroImaging Center, Charité University Hospital, Berlin, Germany; 7 Clinical Affairs, electroCore Medical, Gothenburg, Sweden; 8 Scientific Medical and Clinical Affairs, electroCore LLC, Basking Ridge, USA

## Introduction

Neuromodulatory devices offer additional treatment options to refractory pharmacologic intervention for the treatment of chronic cluster headaches (CH). Clinical experience suggests vagus nerve stimulation (VNS) may be beneficial; however, inherent risks are associated with surgical implantation. As such, a need for the development of a non-invasive VNS (nVNS) modality exists.

## Aim

Evaluate the long-term effects of an nVNS device (gammaCore®) in the extension phase of the Prevention and Acute (PREVA) Treatment of Chronic Cluster Headache study.

## Methods

This randomized, controlled study consisted of a 2-week run-in phase, followed by a 4-week randomized (1:1; nVNS vs standard of care [SoC]) phase, and a 4-week extension phase. During the extension phase, subjects delivered 3 consecutive 90-second stimulations prophylactically twice daily (mandatory, right side only) and optionally at the onset of a CH attack (2 stimulations on headache side, 1 on opposite side) for rescue treatment. Efficacy end points evaluated in the extension phase were mean number of all CH attacks, pain intensity (range: no pain to very severe pain), treatment success rate, and use of rescue medication. Safety was assessed by monitoring the frequency of adverse events.

## Results

A total of 97 subjects, across 10 European sites, were randomized to treatment; 90 entered the extension phase. All data has been collected and will be available for presentation at EHMTIC.

## Conclusion

Findings from PREVA are expected to provide further evidence of the sustained clinical effects and continued safety of nVNS in the treatment of chronic CH.

Abstract submitted on behalf of the PREVA Study Investigators.

